# Antioxidant packaging films: application for sustainable food protection

**DOI:** 10.1016/j.crfs.2025.101222

**Published:** 2025-10-10

**Authors:** Iraj Karimi Sani, Bahram Hassani, Nabil Hussain Rasul, Elahe Mansouri, Hadi Eghbaljoo, Mohammad Kaveh, Dayana Hassani, Mahmood Alizadeh Sani, Arezou Khezerlou, Hassan Gholizadeh, Zahra Salamat Mamakani, Seid Mahdi Jafari

**Affiliations:** a Agricultural Engineering Research Department, West Azerbaijan Agricultural and Natural Resources Research and Education Center, AREEO, Urmia, Iran; b Department of Food Industry, Faculty of Agriculture, Ferdowsi University of Mashhad, Mashhad, Iran; c Department of Food Technology, College of Agricultural Engineering Sciences, Salahaddin University-Erbil, Kurdistan Region, Iraq; d Department of Clinical Nutrition, Faculty of Nutritional Sciences and Dietetics, Tehran University of Medical Sciences, Tehran, Iran; e Division of Food Safety and Hygiene, School of Public Health, Tehran University of Medical Sciences, Tehran, Iran; f Department of Petroleum Engineering, Knowledge University, Erbil, Iraq; g Researcher of Research and Development Unit of Top Tos Campus, Mashhad, Iran; h Department of Food Science and Technology, School of Nutritional Sciences and Dietetics, Tehran University of Medical Sciences, Tehran, Iran; i Nutraceutics Research Center, Tehran University of Medical Sciences, Tehran, Iran; j Nutrition Research Center, Tabriz University of Medical Sciences, Tabriz, Iran; k Department of Food Science and Technology, Ayatollah Amoli Branch, Islamic Azad University, Amol, Mazandaran, Iran; l Department of Food Science and Technology, Technical and Vocational University, Urmia, Iran; m Department of Food Materials and Process Design Engineering, Gorgan University of Agricultural Sciences and Natural Resources, Gorgan, Iran; n Halal Research Center of IRI, Iran Food and Drug Administration, Ministry of Health and Medical Education, Tehran, Iran

**Keywords:** Active packaging, Radical scavenging, Bioactive compounds, Oxidation, Shelf life

## Abstract

Antioxidant packaging films (APFs) have emerged as a transformative result in food preservation, offering biodegradability, cost-effectiveness, and the ability to incorporate bioactive compounds. These films mitigate food spoilage by preventing oxidation and inhibiting enzymatic and non-enzymatic browning, while naturally derived antioxidants enhance their functionality with low toxicity, high nutritional value, and effective antimicrobial and antioxidant properties. Recent advances demonstrate that integrating natural antioxidants, such as polyphenols and flavonoids, significantly improves total phenolic content, free radical scavenging, and overall antioxidant activity of APFs. These films also exhibit great light and moisture barrier properties, mechanical strength, and compatibility with intelligent/active packaging systems. This review investigated novel raw materials, advanced manufacturing techniques, and innovative encapsulation methods for incorporating antioxidants into APFs, highlighting their diverse applications in extending shelf life and ensuring food safety across the food industry, while uniquely addressing critical gaps in scalable, eco-friendly food packaging through cutting-edge developments in sustainable raw materials and enhanced antioxidant delivery for improved film performance.

## Highlights

• Antioxidant packaging films (APFs) effectively inhibit food oxidation.
• APFs suit oxidation-prone foods like oils, meat, dairy, bakery and cereals.
• APFs offer a sustainable alternative to synthetic packaging.
• Advanced encapsulation enhances antioxidant delivery in APFs.
• Novel methods improvement APF antioxidant and barrier properties.

## Graphical abstract

**Figure:**
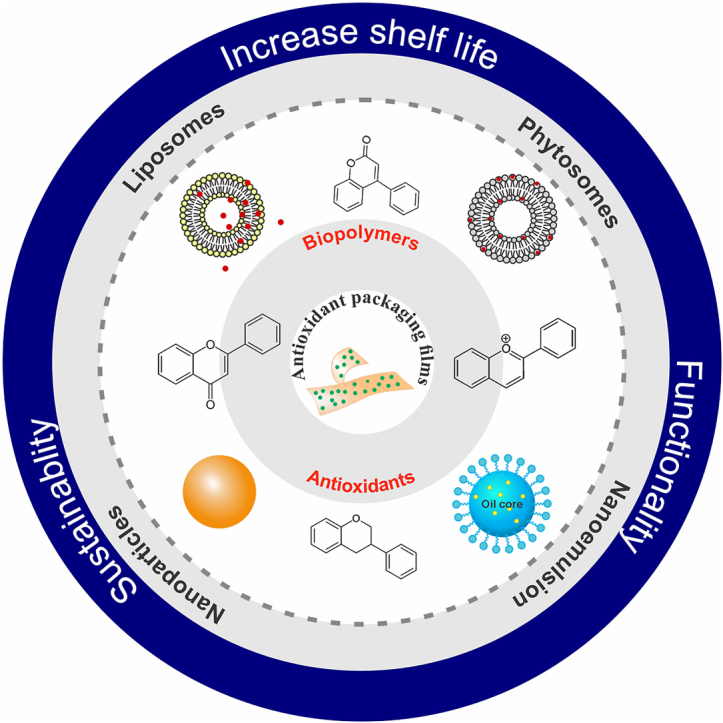

## 1 Introduction

The selection of appropriate packaging materials provide guaranteeing quality, ensuring safety, and extending the shelf life (SL) of food during transportation and storage. Traditionally, food packaging has relied broadly on petroleum-based plastics. These materials offer excellent mechanical properties and moisture resistance but are non-biodegradable, contributing to significant environmental pollution and carbon emissions (Shlush and Davidovich-Pinhas, 2022). This has spurred a critical shift towards exploring renewable resources and biopolymers as sustainable alternatives for food packaging materials (Tan et al., 2021).

Biopolymers, derived from renewable sources such as plants and microorganisms, are emerging as a viable solution. These natural polymers include polysaccharides like starch and cellulose, as well as protein-based polymers such as collagen and whey protein isolate. They can be synthesized into edible films (EFs) that possess desirable properties like toughness, flexibility, and effective barrier characteristics against moisture and gases. While biopolymers are a promising base, their performance can be significantly enhanced through the incorporation of various additives (Deng et al., 2024).

The concept of active and intelligent packaging has gained considerable attention, where EFs are combined with bioactive compounds to provide added functionality. These bioactives such as antioxidants (AOXs), actively interact with the food to preserve its quality. Such additives are crucial for preventing microbial growth, inhibiting oxidation, and maintaining the sensory qualities of food products (Deshmukh and Gaikwad, 2024).

Antioxidants (AOXs) are particularly important in this context, as they protect food from the damaging effects of oxidation, which leads to spoilage and loss of nutritional value. Natural AOXs, such as essential oils (EOs), extracts, pigments, polyphenols, and carotenoids, are preferred over synthetic counterparts due to their safety, consumer acceptance, and additional preservative benefits (Chang et al., 2023). These compounds are integrated into packaging films to create antioxidant packaging films (APFs). APFs not only act as a physical barrier but also actively release AOXs to protect the food from lipid oxidation, thereby extending its SL (Vasile and Baican, 2021).

Despite the potential of innovative APFs in enhancing food preservation through biodegradability, cost-effectiveness, and bioactive compound integration, their widespread adoption faces several challenges. Relatively high cost of production, limited public awareness about their benefits, and difficulties in transitioning from conventional packaging to advanced APF systems delay their scalability and market penetration (Shlush and Davidovich-Pinhas, 2022).

To accelerate the adoption of innovative APFs, cost-effective production methods must be optimized, public awareness increased through targeted education, and modernized integration strategies advanced to facilitate the transition to sustainable, antioxidant-based packaging in the food industry. Establishing industry collaborations will further ease the shift from conventional packaging, developing community considerate and enabling large-scale commercial use to enhance food safety and shelf life.

This review presents an innovative synthesis of knowledge on antioxidant packaging films for sustainable food protection, distinct from the majority of prior reviews that predominantly focus on antimicrobial films (Vieira et al., 2022; Jasrotia et al., 2025; Periyasamy et al., 2025). By offering a unique structure and comprehensive evaluation of antioxidant-based materials and their practical applications, this study covers a critical gap in the literature, addressing an underexplored perspective in food safety and sustainability.

Accordingly, this review article comprehensively studies the raw materials, production methods, and encapsulation techniques for incorporating antioxidants into active packaging films. Its primary novelty deceits in a critical and comparative valuation that focuses specifically on operational and commercialization challenges, highlighting the strengths and weaknesses of each approach. Finally, by providing an in-depth investigation of these films' applications across different food industries, this work aims to guide researchers and manufacturers toward the targeted production of the next generation of sustainable packaging.

## 2 Packaging film (PFs) materials; an overview

The development of PFs incorporating bioactive and functional materials has emerged as a promising avenue for exerting control over food quality, enhancing food safety standards, augmenting nutritional value, and extending SL of food products (Deng et al., 2024). However, certain limitations have been observed, including inadequate physicochemical properties and weak mechanical and structural characteristics of the films. To overcome these challenges, researchers have explored the combination of specific materials and techniques to improve the intermolecular forces between different components in film formulation, leading to enhanced overall performance and properties of the films (Amin et al., 2021). EFs, in particular, are predominantly composed of biodegradable biomaterials such as lipids, polysaccharides, or proteins. To enhance the performance of biodegradable packaging materials, they can be enriched with bioactives such as AOXs, vitamins, etc. (Singh et al., 2022).

Studies investigating the raw materials employed in the production of EFs have highlighted the advantages of protein-based materials due to their nutritional value, relative abundance, film-forming capabilities, and enhanced biodegradability compared to polysaccharide and lipid-based films (Kumar et al., 2022). The incorporation of proteins in EFs leads to the formation of a robust, cohesive, and dense layer with strong viscoelastic properties. The film-forming ability is influenced by molecular characteristics of proteins, including molecular weight, structure, charge, flexibility, and thermal stability. Noteworthy advantages of protein-based films encompass high mechanical properties, favorable physical characteristics, and excellent oxygen barrier effects attributed to the network structure maintained by hydrogen bonds (Liu et al., 2024). However, protein-based films may have limitations in terms of their weak water barrier properties, which can be addressed by incorporating other bio-based materials to reduce moisture sensitivity (Lisitsyn et al., 2021).

Polysaccharides are highly prevalent in EFs production (Zhao et al., 2021). The utilization of polysaccharides offers several advantages, including their abundance, availability, low cost, non-toxic nature, chemical stability, compatibility with processing techniques, thermal processing capabilities, pleasant odor, and lipid barrier properties. Moreover, certain polysaccharides exhibit AOXs and antimicrobial activities, thereby contributing to the extension of food SL. However, a notable limitation lies in the inherent hydrophilicity of polysaccharides, resulting in the production of films with relatively weak water vapor barrier properties. Lipids encompass a diverse range of functional groups, such as monoglycerides, diglycerides, triglycerides, phosphatides, phospholipids, cerebrosides, terpenes, fatty acids, and fatty alcohols. The primary advantage of lipids lies in their hydrophobic nature, which confers their ability to impede moisture transfer and safeguard materials against water vapor. When used as coatings, lipid-based EFs exhibit relatively low permeability and contribute to increased film brightness, reduced moisture loss, and lowered packaging costs (Amin et al., 2021). However, unlike proteins and polysaccharides, lipids alone cannot form a cohesive layer. Due to the absence of numerous repeating units connected by covalent bonds, lipids cannot independently generate a cohesive edible layer. To overcome this limitation, various lipids such as plant oils, plant waxes, animal oils, and EOs (possessing water-repellent, antimicrobial, AOXs, aromatic properties, and rich in volatile compounds like terpenes and terpenoids) are incorporated in solution form into EFs to enhance their properties.

## 3 A brief review of antioxidant compounds

In response to the challenges associated with food preservation and SL, the development of EFs has emerged as a promising solution. In recent years, there has been notable progress in the field, particularly with the exploration of a new generation of active EFs incorporating AOXs (Kumar et al., 2022).

Oxygen and free radicals (oxidation process) play a critical role in food deterioration, significantly impacting quality, safety, and shelf life. Oxygen triggers oxidative reactions, leading to lipid peroxidation, protein degradation, and nutrient loss, which compromise flavor, texture, color, and nutritional value (Wu et al., 2024). Free radicals, generated during oxidation, accelerate these processes by attacking biomolecules, causing rancidity in fats, enzymatic and non-enzymatic browning, and microbial growth. These reactions not only degrade sensory attributes but also reduce the shelf life of products like oils, meats, and dairy. APFs mitigate these effects by scavenging free radicals and limiting oxygen exposure, thereby preserving food quality and extending shelf life (Wu et al., 2024; Zhao et al., 2025). To evaluate AOX_AC_, various assays are employed, which measure the ability of compounds to scavenge or neutralize free radicals. Some commonly used AOX_AC_ assays include the 1,1-diphenyl-2-picrylhydrazyl (DPPH) scavenging, 2,2′-azino-bis (3-ethylbenzothiazoline-6-sulfonic acid) (ABTS) scavenging, nitric oxide (NO) scavenging, deoxyribose, ferric reducing AOXs power (FRAP), hydroxyl scavenging, superoxide scavenging, and total AOX_AC_ assays (El-Shafei et al., 2021). In the following section, some specific AOXs commonly used in the development of EFs are discussed. The effects and benefits of these AOXs in PFs are summarized in Table 1.

**Table 1:** Application of antioxidant materials in APFs.

Film matrix	Bioactive	Key contributions	Ref.
Free form of bioactive compounds			
Corn starch	Rice straw extract	• Amorphous phase extract plasticized the film and offered reinforcement. • TPC (mg GA/100 g film) rose from 0.02 to 0.14 with increasing extract levels 0,4,6,80,4,6,8.	Freitas et al. (2023)
Cassava starch	Purple sweet potato (*Ipomoea batatas* L.) extract	• 15 % extract film showed high pH sensitivity • Color change, MC, WS increased with extract percentage • Swelling property decreased with higher extract levels • Anthocyanin content rose from 78.09 to 0.17 mg/100 g with increasing extract level	Rahmadhia et al. (2022)
Zein	Betalains pigment extract	• Films with extract had smoother surface and highest water repellency • Betalain levels increase led to TPC decrease in UF extract • TPC increased in non-UF sample • No ABTS activity difference between UF and non-UF • DPPH and FRAP activity increased in non-UF	Rodríguez-Félix et al. (2022)
Guar gum/CMC	Litchi peel waste extract	• Covalent interactions and hydrogen bonding were observed between components, showing chemical compatibility • Enhanced UV light barrier properties and DPPH scavenging	Deshmukh et al. (2022)
*Dioscorea zingiberensis* starch	Pennyroyal EOs	• EOs at 1–3 % improved UV protection, transparency, strength, and WVP in films. • Starch films with 35 % EOs showed strong antibacterial activity. • DPPH scavenging increased from 1.21 to 1.76 %, ABTS scavenging from 1.63 to 1.18 %.	Shen et al. (2022)
Fish gelatin/chickpea protein	Black seed EOs and copper sulfide NPs	• Composite films with EOs 0.5−00.5−0 and NPs 0.03−00.03−0 showed improved antimicrobial effects on *E. coli* and *S. aureus*. • The EOs and NPs combination resulted in a 52 % increase in AOX_AC_.	Rasul et al. (2022)
Pectin/nano-chitosan	Fennel EOs and potato peel extract	• 2 % extract and 1.0 mg EOs increased antimicrobial activity • Potato peel extract boosted AOX_AC_ of nano-chitosan and pectin films by 33 % and 35 %	Sadadekar et al. (2023)
Chitosan nano-crystals/chitin	*Curcuma longa* L. EOs and red cabbage extract	• Color changes with EOs exposure to gas and pH solutions • TPC increased from 7.93 to 168.73 mg GA/g film • DPPH scavenging increased from 0.03 to 1.79 in 8 % EOs and 1 % extract samples	Fernández-Marín et al. (2022)
Chitosan	Broken rice extract	• Films with 1–3 % extract had water repellency, improved strength, thermal stability, and barrier properties. • Color changes in films with pH and ammonia exposure were visible. • Extract increased DPPH and ABTS scavenging by around 60 %.	Eze et al. (2022)
Gelatin/CMC	Avocado peel extract	• Extract at 200, 300, and 400 mg/L improved film properties (Moisture content: 12.48 %–11.02 %, and Water solubility: 40.13 %–35.39 %) • Films with extract had better vapor barrier and higher colorimetric values and turbidity • DPPH scavenging increased from 2.02 % to 2.44 %	Vargas-Torrico et al. (2022)

Encapsulated form of bioactive compounds						
Nanocarriers type	Film matrix	Product	Bioactive	Nanocarrier characteristics	Key contributions	Ref.
**Nanoparticles**	Chitosan/poly (vinyl alcohol)/fish gelatin	Rainbow trout	Cinnamaldehyde	Particle size: 370.3 nm ZP = 32.2 mV PDI = 0.166	Sustained and controlled cinnamaldehyde release on trout surface extended SL for 4 days	Hosseini et al. (2022)
	Pectin pulp blackberry	–	Chlorophyll from blackberry leaves within CMC/silica NPs	Particle size: 350 nm ZP = 30.5 mV PDI = 0.74	Stronger antibacterial effect on *E. coli* and *S. aureus*.	Sharifi and Pirsa (2021)
	Chitosan	Grass carp	Proanthocyanidins	Particle size: 293.5 nm ZP = −21.8 mV PDI = 0.254 EE: 37.5 %	Films with NPs show good mechanical, barrier, antioxidant and antibacterial abilities	Yu et al. (2022)
**Nanoliposomes**	Soy protein isolate	Shrimp	Phycocyanin	Particle size: 94.21 nm PDI: 0.221 EE: 83.3 %	Films with encapsulated phycocyanin delayed bacterial spoilage and slow increase in TVB-N and pH	Nami et al. (2024)
	Chitosan/zein	Rainbow trout	*Pulicaria gnaphalodes* (Vent.) Boiss. Extract	PDI = 0.92–0.8 EE = 88.05–49.73	Controlled bioactives release on fish surface extended antimicrobial activity for 14 days.	Mehdizadeh et al. (2021)
	Gelatin	Salmon fish	*Litsea cubeba* EOs	Particle size: 168 nm PDI = 0.250 ZP = 32.14 mV EE = 37.8 %	AOX film exhibited strong antibacterial activity against *V. parahaemolyticus*, delaying spoilage and controlling target bacteria growth.	Cui et al. (2022)
	Chitosan/*Xiolirion ataricum* mucilage	–	*Foeniculum vulgare* extract	Particle size: 57 nm PDI = 0.243 ZP = 17.6 mV EE = 85.2 %	Films with NLPs had superior mechanical, thermal, barrier, antimicrobial properties, and AOX_AC_.	Marand et al. (2023)
**Nanophytosomes**	Carboxymethyl cellulose	Rainbow trout	*Perovskia abrotanoides* Kar. EO Catechin	Particle size: <200 nm PDI = ≤ 0.2 ZP = −34 to −52 mV	• Improved storage stability under refrigerated and gastrointestinal conditions. Enhanced SL	Maleki et al. (2025)
	*Alyssum homolocarpum* seed gum	Chicken meat	*Echinacea purpurea (L.)* extract	Particle size: 329.1 nm PDI = ≤ 0.292 ZP = −31.3 mV	• Films containing 20 % Phytosomal extracts exhibited doubling shelf life (14 days) compared to control films (7 days).	Molaveisi et al. (2022)
**Nanoemulsions**	*Cordia dichotoma* gum	–	Salvia mirzayanii extract	Particle size: 168 nm PDI = 0.296 ZP = 11.2 mV	• Films with NEs show improved thickness, AOX_AC_, antimicrobial properties, contact angle, and EAB	Hasheminya and Dehghannya (2021)
	Pullulan-gelatin	–	Fennel extract	Particle size: 15 nm, PDI = 0.262 ZP = 0.01 mV	• Films demonstrate high density, excellent mechanical properties, water barrier properties, and AOX_AC_	Shen et al. (2021)
	Chitosan/anthocyanidin	–	Cinnamon-perilla EOs	Particle size: 11–1424 nm PDI = 0.19–0.34, ZP = 0.436–7.26 mV	• Water repellency of EOs enhances film properties, including mechanical strength and AOX_AC_	Zhao et al. (2022)
	Cheese whey protein/tamarind starch	Tomato	Thyme EOs	Particle size: 18 nm PDI = 0.34	• NEs contribute to extended SL of tomatoes, improving TS, EAB, and antimicrobial properties	Ghoshal (2022)

### 3.1 Essential oils

EOs, which are lipid-based compounds, have become increasingly popular in the development of EFs due to their beneficial effects, including AOX and antimicrobial properties, as well as their ability to improve the structural, mechanical, and physicochemical properties of the films. Encapsulating EOs within nanoscale surfactant micelles can enhance their desirable properties, since allows for a gradual release of EOs, thereby further improving the properties of the film. Moreover, EOs can be easily incorporated into aqueous polymer solutions without any complications, facilitating the development of films with desirable characteristics (Ghoshal, 2022). EOs are water-repellent concentrated aromatic liquids with strong odors produced as secondary metabolites by various plant species. They are rich in terpenoids, particularly monoterpenes and sesquiterpenes, which can be incorporated into EFs to enhance food preservation. When EOs are added to film formulations, the hydroxyl groups of polymers interact with EOs, resulting in improved water resistance, TS, and elongation at break (EAB) of film. In terms of antimicrobial activity, EOs exhibit water-repellent properties that disrupt the cytoplasmic membranes of bacteria, leading to ion transfer, modification of permeability, and leakage of cellular components. It is worth noting that EOs generally demonstrate greater activity against Gram-negative bacteria compared to Gram-positive ones, which is likely due to the presence of an outer membrane (lipopolysaccharide) in Gram-negative bacteria (Shahidi and Hossain, 2022).

Liu et al., (2021) investigated konjac glucomannan-based EFs loaded with thyme EOs (v/v% 0–1.6). The analysis of the film microstructure revealed that EOs were evenly distributed within the films. As the concentration of EOs increased, the water affinity and TS decreased, while EAB of film and AOX_AC_ increased. The AOX_AC_ was measured in terms of total phenolic content (TPC, approximately 17.5 mg gallic acid eq/g film) and radical scavenging activity (approximately 32.5). EOs-containing films also exhibited specific antibacterial activities against *L. monocytogenes*, *S. aureus*, and *E. coli* O157:H7. The study demonstrated that the incorporation of EOs improved the physical properties, AOX_AC_, and antibacterial activities of pure konjac glucomannan films.

### 3.2 Plant extracts

These plant-based extracts offer a range of advantageous properties, such as providing protection against chronic diseases associated with dietary factors, safeguarding liver health, exhibiting anti-tumor properties, demonstrating AOX_AC_, showcasing antimicrobial properties, exerting anti-diabetic effects, influencing the modulation of the human gut microbiota, promoting the proliferation of beneficial bacteria like *Bifidobacterium* and *Lactobacillus*, and reducing the presence of pathogenic bacteria like *Clostridium histolyticum*, among other effects (Zolfaghari et al., 2023). Incorporation of plant extracts into EFs has been found to enhance their active properties as AOX and antimicrobial compounds, thereby improving the overall quality and extending SL of food products (Hassani et al., 2024). However, it is worth noting that the use of high concentrations of plant extracts in these films can result in undesired outcomes e.g., development of odor, turbidity, and deposition on the films, or higher oxidation in lipid systems. Moreover, the inclusion of plant extracts in films can affect their transparency, as plant extracts are commonly employed for coloration in polymers. Nevertheless, incorporation of plant extracts in EFs can serve as an effective barrier against light, thereby preventing the degradation of ascorbic acid and subsequent browning of food products (Ribeiro et al., 2021).

### 3.3 Pigments

Natural pigments encompass a wide range of bioactives such as carotenoids, flavonoids, anthocyanidins, etc (Eghbaljoo et al., 2023). These pigments serve as crucial secondary metabolites in plants, playing diverse roles throughout their life cycle and exhibiting potent AOX_AC_ (Tavassoli et al., 2024). Their utilization is primarily driven by their inherent properties, as well as their role in determining the visual appeal and perceived quality of products by consumers. Apart from their potent AOX_AC_, they also offer a multitude of health benefits, including anti-cancer, antiviral, antibacterial, anti-inflammatory, anti-allergic, antithrombotic, anti-atherogenic, heart-protective, liver-protective, neuroprotective, anti-malarial, nerve system repair, anti-leishmanial, and anti-aging effects (Lu et al., 2021).

The physical and AOX properties of EFs based on carboxymethyl cellulose (CMC) were investigated by Rojas-Bravo et al. (Duan et al., 2022). These films incorporated red prickly pear (*Opuntia ficus-indica* L. cv. San Martín) peel powder at 0, 1, and 2 %, as well as aqueous extracts at 0, 2, and 4 %. The highest levels of TPC, DPPH scavenging, and reducing power were achieved with the highest concentrations, measuring 542.4 mg gallic acid/100 g, 138.2 mg gallic acid/100 g, and 966.1 mg ascorbic acid/100 g, respectively. Importantly, the mechanical properties of films remained unaffected by the presence of the aqueous extract.

### 3.4 Other bioactives

Bioactives, including curcumin, quercetin, resveratrol, rutin, lutein, and β-carotene have garnered significant attention from researchers due to their diverse physiological functions. However, these bioactives often possess nonpolar chemical structures and exhibit hydrophobic properties. Consequently, they are susceptible to instability when exposed to environmental factors e.g., light and heat, which limits their usage in food formulations (Yuan et al., 2022). In contrast, natural bioactives are characterized by lower toxicity and are more readily accepted by consumers due to their AOX, antimicrobial, coloring, flavoring, and/or nutritional effects (Khedri et al., 2021). Additionally, certain bioactives such as carotenoids, curcumin, and anthocyanins possess strong natural colors that can alter the properties of food materials, making them suitable as natural quality indicators for food products (Chen et al., 2022). In a recent study by Wu et al., (2023), APFs based on starch and gelatin were loaded with resveratrol (0–20 % w/w). The films were prepared using compression molding extrusion. Scanning electron microscopy (SEM) analysis revealed that the films containing resveratrol exhibited higher surface roughness compared to those without resveratrol. Fourier-transform infrared (FTIR) spectroscopy indicated the presence of physical interactions, specifically hydrogen bonding, among starch, gelatin, and resveratrol within the films. The addition of resveratrol led to an increase in film thickness, opacity, and TS, while simultaneously reducing the permeability of the films to water, water vapor, and oxygen. Moreover, the films exhibited higher EAB, surface hydrophobicity, and thermal stability with the addition of resveratrol. Most importantly, the composite films demonstrated excellent AOX_AC_, which was proportional to the concentration of resveratrol. The film containing 20 % resveratrol had the highest oxygen barrier properties and AOX_AC_.

## 4 Encapsulation of antioxidants for loading into PFs

Encapsulation is a process that involves loading bioactives within a protective polymer shell. This shell acts as a barrier, safeguarding the bioactives from external negative effects and facilitating their controlled release in specific environments (Nagar, 2019). The advantages of encapsulation include preserving bioactives against degradation caused by environmental conditions, enhancing the desirable properties of food ingredients, masking unpleasant odors or tastes, preventing incompatibility between encapsulated components, modifying the physical properties of materials, increasing the bioavailability of bioactives, and enabling controlled release, among others (Khezerlou and Jafari, 2020). The choice of an appropriate encapsulation technique depends on various factors, e.g., the nature of the target bioactives, the type of wall materials, and the desired morphology and size of the carriers. Optimizing these factors significantly enhances the encapsulation efficiency (EE). In the context of AOXs, which have the ability to interact with free radicals and terminate undesirable reactions by converting them into harmless products, encapsulation plays a crucial role. AOXs, whether natural or synthetic, often face limitations in terms of weak absorption, challenges in crossing cellular membranes, and potential degradation during delivery. Encapsulation assists in overcoming these limitations by increasing the bioavailability of AOXs. In the following discussion, various carriers (Fig. S1) will be explored. Table 1 provides a partial overview of studies focusing on the free form and encapsulated form of AOXs using various nanocarriers and their subsequent incorporation into PFs.

### 4.1 Nanoparticles (NPs)

NPs have dimensions ranging from 1.0 to 100 nm. Owing to their small size and significantly large surface area, NPs contribute to the improvement of desired properties. Incorporating NPs into EFs can enhance properties such as barrier properties, mechanical strength, heat resistance, and surface characteristics. Furthermore, NPs can offer functionalities such as recyclability, transparency, antimicrobial activity, and UV transmission prevention in EFs. Thus, depending on the specific weaknesses of an EF, adding NPs can enhance its functional and structural properties for food packaging applications. The SL of sweet basil leaves enhanced through the use of a CSNPs-based edible coating containing encapsulated thyme EOs (Hassan et al., 2021).

### 4.2 Nanoliposomes (NLPs)

NLPs are colloidal structures consisting of a bilayer phospholipid membrane, forming spherical lipid vesicles with hydrophilic heads and hydrophobic tails. In an aqueous environment, the hydrophobic groups of phospholipids face the interior core, while the hydrophilic groups face the outer surface. As a result, NLPs can encapsulate both hydrophilic and hydrophobic bioactives, retaining them within the bilayer walls (for hydrophobic molecules) or in the central aqueous phase (for hydrophilic molecules) (Lopez-Polo et al., 2021). NLPs improve the stability and preservation of encapsulated compounds such as polyphenols, EOs, extracts, anthocyanins, and bioactive peptides. Furthermore, NLPs enhance the mechanical strength and water vapor properties of PFs, act as barriers against microorganisms, and extend SL of food products (Homayounpour et al., 2021).

### 4.3 Nanophytosomes (NPYs)

NPYs are formed by the interaction of phosphatidylcholine (or another hydrophilic polar group) and plant extracts in a solvent. In this process, the hydrophilic phosphatidyl moiety completely envelops the hydrophilic phytocholine-choline complexes within the lipid structure (Barani et al., 2021). By incorporating NPYs into PFs, bioactives can be effectively encapsulated. This approach enhances stability, biocompatibility, gastrointestinal absorption, AOX_AC_, antimicrobial activity, and nutrient protection against various processes.

### 4.4 Nanoemulsions (NEs)

NEs are colloidal dispersion systems composed of immiscible liquids, typically stabilized by surfactants (Xia et al., 2021). The reduced droplet size in NEs allows the dominant influence of Brownian motion over gravitational force, resulting in a highly stable system with enhanced resistance to droplet aggregation compared to conventional emulsions. Moreover, NEs possess optical transparency due to the minute droplet size, which falls below the wavelength of light, thereby exhibiting weak light scattering properties. Scientific studies and research have shown that NEs have the capability to improve the WS, stability, performance, and activity of bioactives, particularly lipid-soluble compounds, in both food matrices and digestive environments. NEs, therefore, serve as highly stable colloidal systems for efficient delivery of vitamins, flavorings, AOXs, preservatives, and nutrients (Kong et al., 2022). Additionally, in recent years, NEs-based EFs have emerged as a promising approach in the field of PFs for food products, offering improved quality, safety, performance, and nutritional value.

To conclude, for enhancing the discussion on APFs, carriers are critical to protect antioxidants from environmental degradation (e.g., oxidation, light, or heat), control their release rate, and ensure compatibility with the biopolymer matrix of APFs. Various carriers, such as liposomes, phytosomes, emulsions, or nanoparticles, are selected based on the antioxidant's chemical nature e.g., hydrophilic polyphenols suit polymeric particles, while lipophilic carotenoids pair with lipid-based carriers. The carrier type directly impacts antioxidant effectiveness by enhancing stability, bioavailability, and targeted release, thereby improving functional performance. Additionally, carriers influence film properties, including mechanical strength, barrier properties (e.g., against moisture and light), and bioactive release kinetics, ultimately optimizing APF functionality for extended food shelf life and safety.

## 5 Production of PFs

The production of EFs can be accomplished through various techniques, encompassing wet methods such as casting, dry processes like extrusion, electrospraying, injection molding, thermal forming, compression molding, electrospinning (ES), etc. (Fig. S2). In terms of preparing films for research purposes, the casting method is widely employed due to its simplicity and preference at the lab scale. Moreover, ES method has emerged as a favored technique at the lab scale, demonstrating promising potential in bridging the gap between small-scale research and traditional scaling-up methods, especially for active packaging components sensitive to elevated temperatures. Conversely, extrusion is recognized as a preferred approach for film formation on a commercial scale, catering to the requirements of manufacturers (Westlake et al., 2023). This section will delve into an in-depth examination of various technologies employed in the production of EFs.

### 5.1 Casting

Casting is a simple, cost-effective method for producing EFs on a small scale, with potential for industrial-scale continuous casting (Fig. S2A). The process involves dissolving a biopolymer in a solvent, spreading it onto a surface or mold, and drying it by air or oven to form a film, which is then separated. Film properties depend on solution composition, casting thickness, and drying conditions. Casting requires minimal equipment, enables uniform film structure, and operates at low temperatures, yielding high optical clarity. However, limitations include restricted film shapes, potential toxic solvent retention, protein denaturation, wrinkles, cracks, and long drying times, making it less practical for large-scale production (Ribeiro et al., 2021).

Zhou et al., (2021) investigated carboxymethyl chitosan/pullulan EFs enriched with galangal EOs using the casting method. The compatibility of the composite films was confirmed by FTIR and X-ray diffraction (XRD) analysis, which revealed interactions between the hydroxyl groups of pullulans and carboxymethyl chitosan. The thermal stability of the films was verified through thermogravimetric analysis (TGA) curves. The film containing 8 % EOs demonstrated effective preservation of mango fruits during a 15-day storage at 1–25 °C. Other studies have also designed multifunctional APFs, such as methylcellulose/chitosan nanofibers loaded with zinc oxide NPs, quercetin and natamycin, Zedo gum and CMC formulated with *Lemon verbena*/*Ferulago angulata* extracts for the preservation of raw chicken meat (Razmjoo et al., 2022), and PVA-starch incorporated with coconut shell extract and sepiolite clay as an antioxidant film (Tanwar et al., 2021).

### 5.2 Extrusion

Extrusion is a thermoplastic-based method for producing polymeric films, priduction polymers' thermoplastic by heating a film-forming solution with a plasticizer above its glass transition temperature under low moisture conditions (Fig. S2B). It is environmentally friendly due to minimal solvent use and fewer evaporation stages, making it suitable for commercial applications (Ribeiro et al., 2021). The process involves three zones: feeding, melting, and final heating/output (Eze et al., 2022). Extrusion offers advantages like shorter processing times, lower energy use, enhanced mechanical and optical properties, cost-effectiveness, solvent-free operation, and compatibility with a wide range of temperatures (70–500 °C) and pressures (0–500 bar). However, limitations include restrictions on processing temperature and moisture-resistant materials, high initial equipment costs, and increased maintenance costs (Westlake et al., 2023). The extrusion method, like the casting, has been used in the design of APFs, e.g., PLA/polyolefin elastomer/selenium NPs/triethyl citrate (Zibaei et al., 2023), and starch/gelatin/resveratrol (Wu et al., 2023).

### 5.3 Electrospinning

Electrospinning (ES) is a versatile non-mechanical technique for producing nanofibers (NFs) from biopolymer solutions using a high electric field at room temperature and atmospheric pressure (Fig. S2C). It yields NFs with high porosity and surface-to-volume ratio. Common ES methods include physical absorption, covalent fixation, coaxial ES, and hybrid approaches. Benefits include enhanced molecular orientation, extensive porosity, suitable morphology, micro-to nano-diameter, adaptability, and efficient bioactive encapsulation (Chawla et al., 2021). This method has been widely used for APFs such as antioxidant peptide-loaded electrospun chitosan-flaxseed mucilage NFs for sustained release of *Ziziphora clinopodioides* EO and sesame oil (Karami et al., 2021).

## 6 Impact of antioxidant compounds on packaging film properties

The incorporation of AOXs into PFs can have significant effects on various aspects of the films. Bioactives, through their interactions (both covalent and non-covalent) with reactive groups present in polymer chains, have the potential to enhance the structural, physical, chemical, optical, mechanical, and barrier properties of PFs. Furthermore, certain bioactives can improve the antibacterial or AOX properties of EFs, ultimately leading to an extended SL for food products.

### 6.1 Physical properties

The physical properties of biopolymers play a crucial role in determining the characteristics of films and coatings. The incorporation of different levels of bioactives leads to the formation of EFs with varying thicknesses (Gupta et al., 2022). As example, the incorporation of curcumin into starch films increased their thickness from 0.019 to 0.023 mm (Mali and Pandey, 2024). WS is another important parameter in selecting films for specific applications. The presence of hydrophobic and insoluble components in bioactives reduces WS of the films (Kong et al., 2022). In one study, the addition of Date palm pit extract led to a reduction in the WS of alginate films from around 86.98 to 54.60 % (Khwaldia et al., 2023).

### 6.2 Mechanical properties

The mechanical properties, including TS, EAB, and Young's modulus (YM), are essential functional characteristics of food PFs. These properties are influenced by the intermolecular and intramolecular interactions within the polymer matrix. The incorporation of bioactives can modify the molecular interactions among biopolymer molecules in EFs, leading to notable effects on their structure and mechanical properties (Chen et al., 2022). The adding a curcumin to starch films increased the TS (from 17 to 29 MPa), while reduced EAB (from 27 to 19 %) compared to the control film (Mali and Pandey, 2024). In another study, adding of epicatechin gallate increased the TS (from 27.94 to 36.20 MPa) and EAB (from 38.36 to 56.40 %) values of chitosan films (Yong et al., 2024).

### 6.3 Barrier properties

The control of moisture and gas transfer is crucial for the functionality of PFs in food packaging. The permeability to water vapor (WVP) and oxygen (OP) of PFs, a key measure of their moisture and oxygen barrier properties, is influenced by the incorporation of bioactives (Firouz et al., 2021). These bioactives can act as plasticizers or cross-linking agents, and their hydrophilic or hydrophobic properties can alter the film's barrier property. By chemically bonding with the polymer matrix, bioactives can improve the film's structural integrity, thus reducing its permeability to water vapor and gases (Chen et al., 2022). Thr integration of a curcumin/β-cyclodextrin complex to sodium alginate films reduced the OP (from 1.72 to 1.06 × 10^−3^ g/(m^2^·s)) and WVP (from 9.02 to 6.50 × 10^−11^ g/m·s·Pa) (Shi et al., 2025). In other work, the addition of different active compounds (zinc oxide nanoparticles, quercetin and natamycin) led to a reduce in the WVP (from 6 to 1.85 × 10− 10 g m/m^2^.s.Pa) of methylcellulose and chitosan nanofibers films (Sani et al., 2023).

### 6.4 Optical properties

The optical properties, including color and transparency, hold significant importance in PFs as they contribute to the visual appeal and consumer acceptance of the packaging. Bioactives incorporated into PFs can possess inherent colors or exhibit varying degrees of brightness. The color of AOXs, in particular, can serve as a barrier against specific wavelengths, such as UV light, thereby maximizing SL of packaged foods. Hence, assessing the optical properties of PFs is crucial. The chitosan films incorporated with caffeic acid–grafted inulin showed notable UV absorption ability, with a UVC and UVB shielding rate of 0 % (Tan et al., 2025). The addition of *rhododendron arboreum* Sm. Anthocyanin to starch, chitosan, and polyvinyl alcohol films could provide reduction in UV light barrier (Shahi et al., 2025).

### 6.5 Thermal stability

The evaluation of thermal properties of PFs involves two primary methods: TGA to assess the impact of incorporating bioactives on thermal stability, and differential scanning calorimetry (DSC) to analyze heat transfer effects (Santana and Bonomo, 2024). TGA reveals that even the addition of bioactives in small amounts can reduce the thermal stability of the resulting films, and higher concentrations of these compounds further decreases thermal stability. DSC analysis indicates that the presence of bioactives induces changes in the thermal behavior of the films, such as degradation occurring at lower temperatures and variations in *T*_*g*_, crystalline melting temperature (*T*_*m*_), and enthalpy changes (*ΔH*) (Shah et al., 2024). As an example, the thermal stability of tragacanth gum and carboxymethyl chitosan films increased with β-cyclodextrin-quercetin complex by reduction of rate of weight loss (Liu et al., 2025).

### 6.6 Antioxidant properties

The incorporation of AOXs to EFs significantly boosts their ability to fight oxidation, prevent browning, and protect nutrients. The AOX_AC_ of these films is influenced by the type of bioactive compound, the film-forming material, and the encapsulation technique. Natural ingredients like polyphenols, essential oils, and carotenoids are often used for their inherent AOX_AC_, which they achieve by neutralizing harmful molecules and stabilizing free radicals. The selection of the right bioactive is crucial, as the film's antioxidant capacity is directly tied to the concentration of the bioactive trapped within it. The inclusion of *Tribulus terrestris* extract in chitosan/oxidized microcrystalline cellulose films yielded a ∼4 and ∼3.7 fold increment in ABTS^•+^ and DPPH^•^ scavenging (Doğan Ulu et al., 2025). In another work, the addition of onion peel (0–20 wt%) was reported to increase the ABTS^•+^ scavenging activity (4–93 %) of starch-based bioplastics (Vallejo et al., 2025).

## 7 Application of APFs in food products

APFs are used in the food industry to improve the shelf life and safety of food products, especially ready-to-eat meals, meats, dairy, bakery, fruits, and vegetables. These films work by directly interacting with the food to reduce the growth of microorganisms and protect against spoilage factors like oxidative rancidity and enzymatic browning. Some APFs even have self-sterilizing properties, which helps maintain the quality and extend the shelf life of packaged foods. Table 2 has been summarized the application of bioactive films in different food products.

**Table 2:** Application of APFs in different food products.

Food type	Film matrix	Bioactive	Product	Key contributions	Ref.
**Meat and meat products**	Chitosan/collagen	*Pleurotus ostreatus* polysaccharide-Epigallocatechin gallate conjugates and ε-Polylysine	Pork	• The composite film slowed lipid oxidation and inhibited microbial growth. • They increased the SL of pork for 5 days.	Niu et al. (2025)
	Potato starch	Thyme oil	Beef	• pH levels slightly increased from 6.21 to 6.76 b y day 8 for control meat and from 6.21 to 6.70 b y day 12 for active film-packaged meat. • Active film packaging resulted in lower microbial population compared to the control sample.	Yuan et al. (2021)
	Gelatin and isolated chickpea protein	Black seed EOs	Beef	• The microbial population decreases from 7 to 37 in the control beef sample and from 7 to 19 in the beef covered with the active film over a storage period of 14 days, resulting in higher SL.	Rasul et al. (2022)
**Dairy products**	Sodium alginate	Basil, rosemary, and mint EOs	Local cheese	• Weight loss decreased from 19.31 % to 15.30 % in the cheese with the active film. • The active film preserved protein and fat stability. • It inhibited the growth of Salmonella and fungi.	Mahcene et al. (2021)
	Carrageenan	Aloe vera extract	Ice cream	• Microbial count, free fatty acids, and TBA decreased in the ice cream. • PV reduced from 3.4 to 1.07 meq/kg in ice cream with plant extract film.	Mahajan et al. (2021)
	Polyethylene	Thymol and linalool	Mozzarella cheese	• Fungal and yeast growth decreased in treated sample. *S. aureus* and *E. coli* reduced during storage with active film.	Chang et al. (2021)
**Bakery products**	Poly lactic acid and polybutylene terephthalate	Carvacrol	Butter cake and white bread	• Inhibitory effects on *Penicillium* and *Rhizopus* fungi, extending bakery product SL.	Klinmalai et al. (2021)
	Corn starch-bean protein	*d*-Limonene	Rice flour cake	• Cake SL extended by 2–4 days, enhancing sensory attributes.	Luo et al. (2022)
	Poly vinyl acetate-gelatin	*Heracleum persicum* EOs	Pita bread	• Reduced total microbial count in bread coated with active film.	Salimkhaniyan et al. (2021)
	Poly hydroxy butyrate	Thyme EOs	White bread	• Extended bread SL to 5 days, reduced fungal count.	Qian et al. (2021)
	Corn starch and pectin	Turmeric EOs	Bread	• Absence of microbial contamination over 9 weeks.	Araújo et al. (2023)
	Cellulose acetate	Oregano EOs	Hamburger bun	• Increased bread SL to 12–27 days, improved consumer acceptance.	Fernandes et al. (2022)
**Fruits and vegetables**	Carrageenan-chitosan	Extract of broccoli, sweet potato, and red cabbage	Apple	• Superior AOX_AC_ and TPC in coated apples compared to chitosan-coated and co ntrol samples. Carrageenan film showed the highest AOX_AC_ at 11.97 % after 7 days.	Jancikova et al. (2021)
	Zein	Thyme EOs	Strawberry	• Coated strawberries had reduced bacterial and yeast counts, preserved TPC, AOX_AC_, and acidity during 15-day storage.	Ansarifar and Moradinezhad (2021)
	Potato starch	Fennel EOs	Fresh pistachio	• Pistachios with film had lower weight loss, microbial growth, and improved sensory properties, fat content, and moisture retention. Fungal growth decreased significantly.	Babapour et al. (2022)
	Chitosan-starch	Star anise EOs	Apple	• Lower respiration rate, weight loss, and preservation of firmness and skin brightness during storage.	Long et al. (2022)

### 7.1 Meat and meat products

**Fig. 1:**
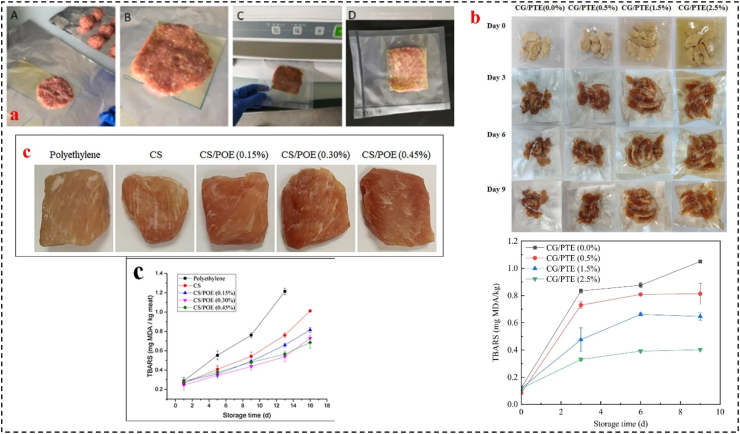
a) Ground beef meat packaging with two cellulose based systems (Barzan et al., 2024), Elsevier, Open access**, b)** Photographs and TBARS results of chicken jerky packaged in cassia gum/PTE films during 9 days of storage (Wei et al., 2024), MDPI, Open access and **c)** Visual image of chilled meat packed with chitosan-starch/*Portulaca oleracea* extract film after 6 days storage, and TBARS results. Adapted from Ref (Fan et al., 2023). with license number 6117471405125.

The application of APFs in meat and meat products is crucial due to the presence of nutrients that can support microbial growth and the susceptibility of unsaturated fatty acids to lipid oxidation. Microbial contamination and lipid oxidation are major challenges in meat preservation, and various factors such as pH, water activity, nutrient composition, oxygen, light, and temperature can influence these reactions. Therefore, it is crucial to control these factors to extend SL of meat products (Alizadeh Sani et al., 2024). Many studies have demonstrated the effectiveness of EFs containing different bioactives in inhibiting the growth of pathogenic microorganisms, lipid oxidation, and protein hydrolysis in meat products, thereby extending their SL. For example, Ningrum et al. investigated gelatin-based films containing eucalyptus EOs and their impact on meat. His edible films applied on beef can maintain the texture and color of up to Day 3 b y inhibiting oxidation and microbial activity (Ningrum et al., 2021). In other work, Barzan et al. (2024) evaluated the potential of moringa and grape macerates extracts at 5 % concentrations to create two types of cellulose-based antioxidant food packaging for preservation of ground beef from oxidative damage (Fig. 1a). These bio-based packaging showed substantial in vitro free radical scavenging activity (50 % antioxidant power) and significantly inhibited lipid oxidation in ground beef by at least 50 % over during 16 days of storage, as evidenced by both indirect thiobarbituric acid reactive substance (TBARS) analysis and direct in situ Raman spectroscopic measurements (Barzan et al., 2024). As another example, Wei et al. (2024) prepared films by combining cassia gum with varying concentrations of partridge tea extract (PTE; 0–2.5 %) (Fig. 1b). The film containing 2.5 % PTE exhibited excellent antioxidant potential (46.88 % DPPH radical scavenging activity) after a 50-fold dilution. When used to package chicken jerk, the CG/PTE films effectively inhibited lipid oxidation. Compared to the control (1.05 mg MDA/kg), the film containing 2.5 % PTE significantly reduced the formation of TBARS (0.402 mg MDA/kg) in chicken jerk after 9 days of storage (Wei et al., 2024). According to study of Fan et al. (2023), the chitosan-starch films containing 0.45 % *Portulaca oleracea* extract significantly decreased TBARS values (28.7 % reduction) in pork meat during 16 days storage at 4 °C (Fig. 1c). These films effectively inhibit lipid oxidation in pork meat, resulting in a significant extension of shelf-life, due to potent antioxidant activity of *Portulaca oleracea* extract (Fan et al., 2023).

### 7.2 Dairy products

**Fig. 2:**
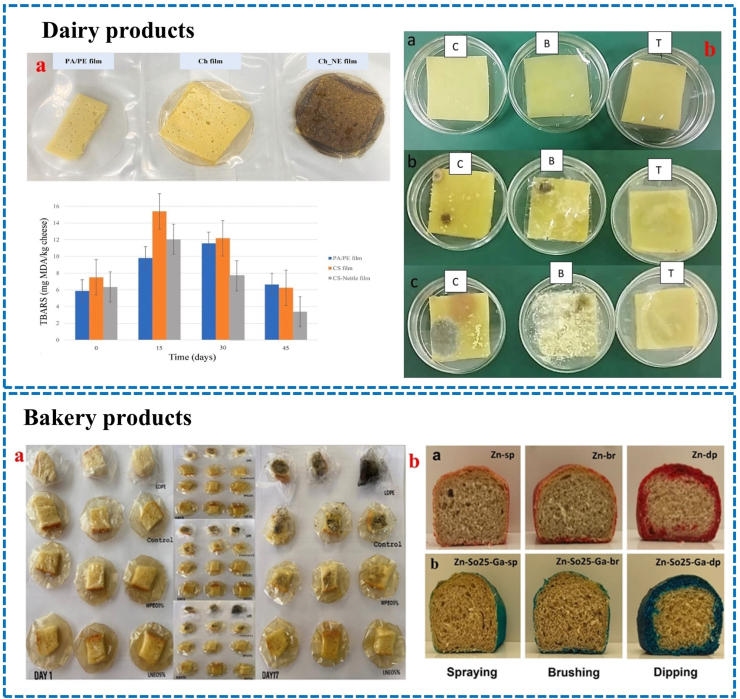
Dairy products: a) visual image of Havarti cheese with slice separator film and TBARS results. Adapted from Ref. (Flórez et al., 2023), with license number 6117450940445, and **b**) Storage results for fruit at different times for various treatments (a: 0, b: 2 weeks, c: 4 weeks; C: unpacked, B: packed with film A, T: packed with film A1) (Rehim et al., 2023), Nature, Open access. **Bakery products: a**) Actual view of white bread slices packed with SPI films (Hosseiniyeh et al., 2024), Wiley, Open access, and **b**) visual images of wheat bread loaf cross-sections coated with (a) zein and (b) zein-sunflower oil (25 %) coatings. Adapted from Ref (Mouzakitis et al., 2022). with license number 6117460527617.

There's a growing global demand for dairy products, but they're highly susceptible to spoilage and undesired changes caused by various external factors, e.g., microorganisms, oxygen, moisture, light, and mechanical forces. This leads to issues such as microbial contamination, oxidation, and changes in flavor and color, which significantly affect the quality of dairy products (Chawla et al., 2021). To address these challenges, the utilization of active EFs and coatings that are environmentally friendly has emerged as a promising approach. Cheese, as a dairy product, is a notable example where EFs and coatings have been employed to mitigate quality deterioration during storage (Ribeiro et al., 2021). For example, Flórez et al. (2023) assayed how chitosan film with nettle extract influences on the oxidative quality of Havarti cheese during storage for 45 days at 5 °C (Fig. 2a). They demonstrated that the film separator effectively suppressed lipid oxidation in cheese during 45 days storage, as evidenced by a significant 56 % reduction in TBARS compared to the control group (Flórez et al., 2023). To effectively preserve the quality of cheddar cheese, Abdel Rehim et al. (2023), developed a film based on polyvinyl alcohol with *Lepidium sativum* extract and hyperbranched polyamide amine. The oxidation ability of extracted fat from the cheese samples ranged from 0.40 to 0.98, confirming the films' ability to resist lipid oxidation (Fig. 2b). Furthermore, the active films effectively inhibited trans-fat formation in the cheese, demonstrating their multifunctional role as antioxidant, antimicrobial, and food-preserving packaging materials. Cheese samples packaged with the active films exhibited significantly extended shelf-life, maintaining quality for up to 4 weeks (Rehim et al., 2023).

### 7.3 Bakery products

Baked and extruded products possess a brittle texture primarily due to their low MC. However, when exposed to higher relative humidity conditions during storage, these products can experience a loss of crispiness due to increased MC (Bizymis and Tzia, 2022). Microbial contamination in baked products is influenced by food composition, moisture, oxygen content, water activity, pH, and additives. Oxidative rancidity can generate free radicals and peroxides, leading to degradation of certain vitamins and proteins (San et al., 2022). PFs used on baked goods offer multiple benefits: reduce oxygen exchange, minimize water vapor permeability, prevent fat/oil leakage, protect against light, provide physical and mechanical protection, and help ensure an adequate Hosseiniyeh et al. (2024) evaluated the impact of incorporating lecithin-emulsified black seed oil nanoemulsions (LNEO) and whey protein isolate-stabilized Pickering emulsions (WPEO) into soy protein isolate (SPI) films on the quality of bread slices for 17 days (Fig. 2a). Bread slices packaged with LNEO-incorporated SPI films demonstrated the most favorable sensory attributes and color retention. Notably, bread slices wrapped in SPI films containing 5 % LNEO exhibited no mold growth throughout the entire 17-day storage period. In contrast, bread slices stored in low-density polyethylene bags began to show signs of spoilage by the 6th day (Hosseiniyeh et al., 2024). According to results of Mouzakitis et al. (2022), wheat bread coated with zein-sunflower oil (5 and 25 %) solution by brushing or spraying showed reduced moisture loss from the inner part (crumb) to the outer part (crust) during 4 days of storage at 25 °C (Fig. 2b). The slow rate and less staling observed in bread coated with a coating solution containing 25 % sunflower oil. Bread coated by brushing showed less starch re-ordering and had increased protein aggregation in the crumb, likely due to ethanol evaporation. Sprayed bread was generally preferred due to fewer off-flavors (Mouzakitis et al., 2022).

### 7.4 Fruits and vegetables

**Fig. 3:**
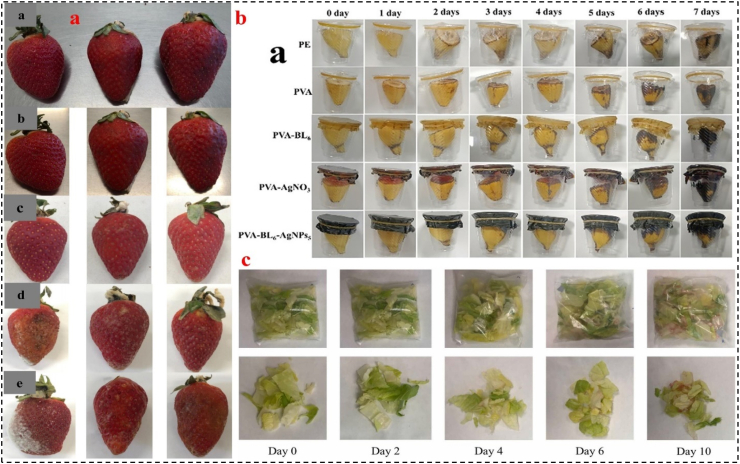
a) Storage results for strawberries at different times (a: 0; b: 4; c: 8, d: 12, and e: 16 day) for various treatments (left to right, control group, glycerol loquat and sorbitol loquat films) Adapted from Ref (Costa et al., 2023). with license number 6117460776901, **b)** Storage results for bananas at different times (7 day) for various PFs. Adapted from Ref (Yang et al., 2024). with license number 6117461066254, and **c)** visual appearance of the fresh-cut lettuce packaged in 80:20 PL A:Ecoflex® + 0.5 % LCNF. Adapted from Ref (Bascón-Villegas et al., 2022). with license number 6117470067201.

The short SL and susceptibility of fresh fruits and vegetables to decay during storage pose significant challenges. The loss of nutrients, water transpiration, and the growth of spoilage microorganisms during preservation negatively affect the visual appearance and taste of these perishable items. To address these issues, the utilization of EFs as environmentally friendly packaging materials offers noteworthy advantages in extending SL of fruits and vegetables (Xie et al., 2025). To address these issues, the utilization of EFs as environmentally friendly packaging materials offers noteworthy advantages in extending SL of fruits and vegetables. For instance, Khalifa et al. evaluated the impact of a chitosan-based film incorporating olive leaf extract on apples. The uncovered apples exhibited higher levels of decay and weight loss compared to the covered samples. Moreover, adding the extract to the films resulted in a gradual reduction of AOXs, flavonoids, and phenolics in the fruit. Similarly, Zhang et al. developed a chitosan-based film containing banana peel extract which led to lower respiration, weight loss, and higher firmness in the covered apple samples compared to those covered with the film alone. Furthermore, it resulted in a remarkable 35 % increase in AOX_AC_. For example, Costa et al. (2023) investigated the effect of loquat seed starch antioxidant coatings on quality of strawberries at 4 °C for 16 days (Fig. 3a). Loquat seed starch films significantly reduced decay rates, leading to enhanced fruit firmness, color retention, and reduced weight loss, extending shelf life to 16 days under refrigeration. The coatings effectively preserved ascorbic acid and total phenolics, likely due to the inherent bioactivity of the modified starch, which inhibited fungal and microbial growth (Costa et al., 2023). In another study, studied the effects of polyvinyl alcohol (PVA) films incorporated with black liquor (BL; 1, 2, 4, 6, 8, or 10 %) and silver nanoparticles (AgNPs; 1, 3, 5, 7, 9, or 10 g/L) on banana quality that stored at room temperature for 7 days (Fig. 3b). The group packaged with PVA-BL6-AgNPs5 films exhibited the least browning after 3 days, minimal weight loss, retained visible yellow skin, and revealed no signs of softening or rotting after 7 days, indicating effective protection against microbial invasion and moisture loss (Yang et al., 2024). Bascón-Villegas et al. (2022) integrated the different amounts of lignocellulose nanofibers (LCNF) derived from enzymatically treated wheat straw waste into a polylactic acid (PLA) and polybutylene adipate-co-terephthalate (Ecoflex®) matrix to develop an antioxidant film for preservation of fresh-cut lettuce during storage at 4 °C for 10 days (Fig. 3c). The lettuce packaged in LCNF-reinforced films maintained microbiological and sensory quality levels comparable to those observed with the commercial packaging (Bascón-Villegas et al., 2022).

### 7.5 Other food products

**Fig. 4:**
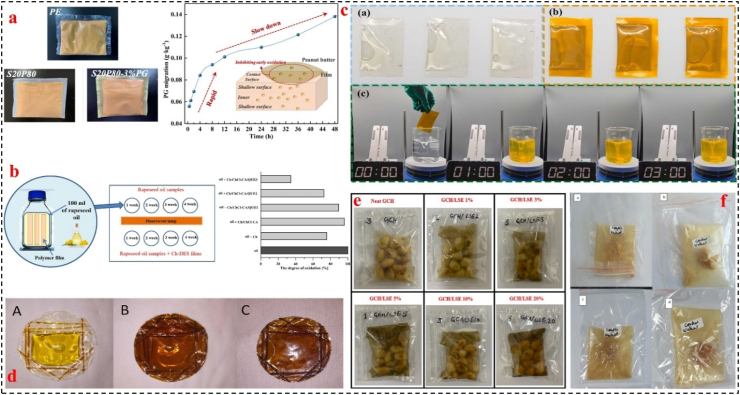
a) The visual properties of peanut butter with PE, S20P80, and S20P80–3 %PG films and PG migration results. Adapted from Ref (Gao et al., 2024). with license number 6117470419870**, b)** Arrangement of oil sample bottle circles in the incubator relative to the fluorescent lamp's position and degree of oxidation of oil results (Jakubowska et al., 2023), Elsevier, Open access**, c)** Heat-sealed pouches with soybean oil (a) SG and (b) SG/Curcumin 0.20 films (c) dissolution test for the SG/Cur 0.20 pouches of soybean oil. Adapted from Ref (Dong et al., 2023). with license number 6117470662597**, d)** Pouches filled with olive oil: (A) chitosan/gelatin film; (B) chitosan/gelatin with 0.5 % gallic acid, and (C) chitosan/gelatin with 2.0 % gallic acid. Adapted from Ref (Kurek et al., 2024). with license number 6117470823752**, e)** visual photo of roasted peanuts packaged in sachets made from GG/CMC-based film containing halloysite nanotube and different concertation of LSWE. Adapted from Ref (Deshmukh et al., 2022). with license number 6117470957804**,** and **f)** Visual image of walnut packed with active film (a, c) and control film (b, d), (upper row; 0 day, lower row; 8 days). Adapted from Ref (Kumar et al., 2024). with license number 6117471108825**.**

For instance, Gao et al. (2024) developed a poly (butylene adipate-co-terephthalate) (PBAT)/starch-based antioxidant films with propyl gallate (PG) and used it in peanut butter preservation. The starch/PBAT (20:80) films containing 3 % PG effectively suppressed lipid oxidation, keeping the peroxide value below the limit (0.25 g/100 g) throughout the 300-day storage period (Fig. 4a). The controlled release of PG from the starch matrix, coupled with the low oxygen and UV barrier properties of the film, significantly enhanced the shelf-life and quality of the packaged peanut butter (Gao et al., 2024). In another study, Jakubowska et al. (2023) investigated the effect of chitosan films containing choline chloride and citric acid (deep eutectic solvent) and quercetin (1 and 3 %) in protecting rapeseed oil from oxidation (Fig. 4b). These films significantly inhibited secondary lipid oxidation processes in rapeseed oil, thereby improving its antioxidant stability during accelerated storage conditions (28 days at 40 °C). Only rapeseed oil protected by 3 % quercetin film maintained the total oxidation index value (9.69) below the acceptable limit (<10) after 4 weeks of storage (Jakubowska et al., 2023). Dong et al. (2023) prepared a soluble soybean polysaccharide and gelatin (SG) film with curcumin and used it to create a pouch for storing soybean oil at 50 ± 1 °C for a period of 7 days (Fig. 4c). Soybean oil packaged within the SG/curcumin 0.2 pouch exhibited the lowest PV (12.8 meq/kg). The curcumin in the film helped to slow down the oxidation of the soybean oil during storage (Dong et al., 2023). Similarly, in another work by Kurek et al. (2024), olive oil was pouched with chitosan, gelatin, and gallic acid and declared the olive oil samples had low level of volatile compounds produced by oxidative process (Fig. 4d) (Kurek et al., 2024). As another example, Deshmukh et al. (2022) reported the guar gum/carboxymethyl cellulose (GG/CMC)-based film enriched with 20 % litchi shell waste extract (LSWE) showed lower TBARS (0.97 mg MDA/kg) values compared to the sample packed with guar gum/carboxymethyl cellulose films (5.21 mg MDA/kg) after 8 days (Fig. 4e) (Deshmukh et al., 2022). Kumar et al. (2024), reported the oxidative stability of walnut packed in chitosan and *Tulsi-Ajwain* EOs films ameliorate after 8 days (as seen in Fig. 4f) (Kumar et al., 2024).

## 8 Challenges and opportunities

Antioxidants in APFs can prevent oxidative rancidity and prolong the freshness of perishable items. However, their use in APFs is not without challenges. One of the primary challenges associated with using antioxidants in APFs is ensuring their effectiveness over time. The stability of antioxidants can be influenced by several factors, including temperature, light exposure, and humidity. For instance, some antioxidants may degrade under high temperatures or UV light, reducing their ability to oppose oxidative processes (Duan et al., 2022). Moreover, the diffusion of antioxidants from APFs into the food product can vary significantly based on the polymer matrix used. This variability can lead to inconsistent antioxidant concentrations, leaving some areas of the food inadequately protected. Studies have shown that the release rate of antioxidants can be difficult to predict, complicating the formulation of effective packaging solutions.

Another significant challenge is the compatibility of antioxidants with different packaging materials. Many commonly used polymers have limited affinity for certain antioxidants, which can lead to poor dispersion and uneven distribution within APFs. This incompatibility can fail to achieve the desired protective effects (Rangaraj et al., 2021). Furthermore, the incorporation of antioxidants may alter the physical properties of APFs, including their mechanical strength, flexibility, and barrier properties. Striking a balance between antioxidant efficacy and maintaining the desired properties of the packaging material is a complex task that requires extensive research and development.

## 9 Conclusion and future outlook

In an era where food preservation is increasingly crucial to minimize waste and extend SL, the development of APFs has emerged as a significant innovation. These advanced materials not only serve as barriers to external contaminants but also actively contribute to maintaining the quality and safety of food products. APFs are designed to mitigate oxidative degradation, which is a primary cause of food spoilage. Oxidation can lead to undesirable changes in taste, color, and nutritional value. These films are typically composed of biodegradable polymers infused with natural or synthetic antioxidants. Common polymers which are modified to enhance their barrier properties and mechanical strength. The efficacy of APFs joints on several critical properties including improved barrier properties, mechanical strength, release kinetics, and biodegradability. The applications of these innovative films are diverse and growing. They are particularly beneficial in the packaging of perishable items such as fruits, vegetables, meat, and dairy products. Therefore, these active packages are a promising development in the food packaging industry that can reduce the amount of food waste by inhibiting oxidation and increase the safety and quality of products.

## Author's contribution

The authors contributed to writing the initial draft of various sections of the article. Iraj Karimi-Sani, Mahmood Alizadeh Sani and Seid Mahdi Jafari edited and reviewed the final article.

## Credit author statement

Iraj Karimi Sani: Formal analysis, Investigation, Writing – original draft, review & editing. Bahram Hassani: Formal analysis, Investigation, Writing – original draft. Nabil Hussain Rasul: Formal analysis, Investigation. Elahe Mansouri: Funding acquisition, Project administration, Writing – review & editing. Hadi Eghbaljoo: Funding acquisition, Project administration, Writing – review & editing. Mohammad Kaveh: Funding acquisition, Project administration, Writing – review & editing. Dayana Hassani: Formal analysis, Investigation, Writing – original draft. Mahmood Alizadeh Sani: Funding acquisition, Project administration, Writing – review & editing. Arezou Khezerlou: Formal analysis, Investigation, Writing – original draft. Hassan Gholizadeh: Funding acquisition, Project administration, Writing – review & editing. Zahra Salamat Mamakani: Formal analysis, Investigation, Writing – original draft, review & editing. Seid Mahdi Jafari: Funding acquisition, Project administration, Writing – review & editing.

## Declaration of competing interest

The authors declare that they have no known competing financial interests or personal relationships that could have appeared to influence the work reported in this paper.
