# Supplementary material for: Antioxidant packaging films: application for sustainable food protection
Source: Curr Res Food Sci. 2025 Oct 10;11:101222. doi: 10.1016/j.crfs.2025.101222 (PMC12547824; doi:10.1016/j.crfs.2025.101222)
Supplement: Multimedia component 1 [file mmc1.docx]

**Antioxidant packaging films: innovative sustainable food protection**

**Iraj Karimi Sani^a1^*, Bahram Hassani*^b1^, Nabil Hussain Rasul^c^, Elahe Mansouri^d^, Hadi Eghbaljoo^e1^ , Mohammad Kaveh^f^, Dayana Hassani ^g1^, Mahmood Alizadeh Sani ^h^,*, Arezou Khezerlou^i^, Hassan Gholizadeh^j^ , Zahra Salamat Mamakani^k^, Seid Mahdi Jafari^L,m^ ***

^a^ Agricultural Engineering Research Department, West Azerbaijan Agricultural and Natural Resources Research and Education Center, AREEO, Urmia, Iran.

^b^ Department of Food Industry, Faculty of Agriculture, Ferdowsi University of Mashhad, Mashhad, Iran.

^c^Department of Food Technology, College of Agricultural Engineering Sciences, Salahaddin University-Erbil, Kurdistan Region, Iraq.

^d^ Department of Clinical Nutrition, Faculty of Nutritional Sciences and Dietetics, Tehran University of Medical Sciences, Tehran, Iran.

^e^ Division of Food Safety and Hygiene, School of Public Health, Tehran University of Medical Sciences, Tehran, Iran.

^f^ Department of Petroleum Engineering, Knowledge University, Erbil, Iraq.

^g^ Researcher of research and development unit of Top Tos Campus, Mashhad, Iran.

^h^ Department of Food Science and Technology, School of Nutritional Sciences and Dietetics, Tehran University of Medical Sciences, Tehran, Iran.

^i^ Nutrition Research Center, Tabriz University of Medical Sciences, Tabriz, Iran.

^j^ Department of Food Science and Technology, Ayatollah Amoli Branch, Islamic Azad University, Amol, Mazandaran, Iran

^k^Department of Food Science and Technology, Technical and Vocational University, Urmia, Iran.

^L^Department of Food Materials and Process Design Engineering, Gorgan University of Agricultural Sciences and Natural Resources, Gorgan, Iran.

^m^ Halal Research Center of IRI, Iran Food and Drug Administration, Ministry of Health and Medical Education, Tehran, Iran.

**^1^ Equal to the first author**

***Corresponding authors;**

Iraj Karimi Sani ([eng.irajkarimi@gmail.com](mailto:eng.irajkarimi@gmail.com)), Seid Mahdi Jafari ([smjafari@gau.ac.ir](mailto:smjafari@gau.ac.ir)), Mahmood Alizadeh Sani ([saniam7670@gmail.com](mailto:saniam7670@gmail.com)), Bahram Hassani (Bahram.Hassani@alumni.um.ac.ir)


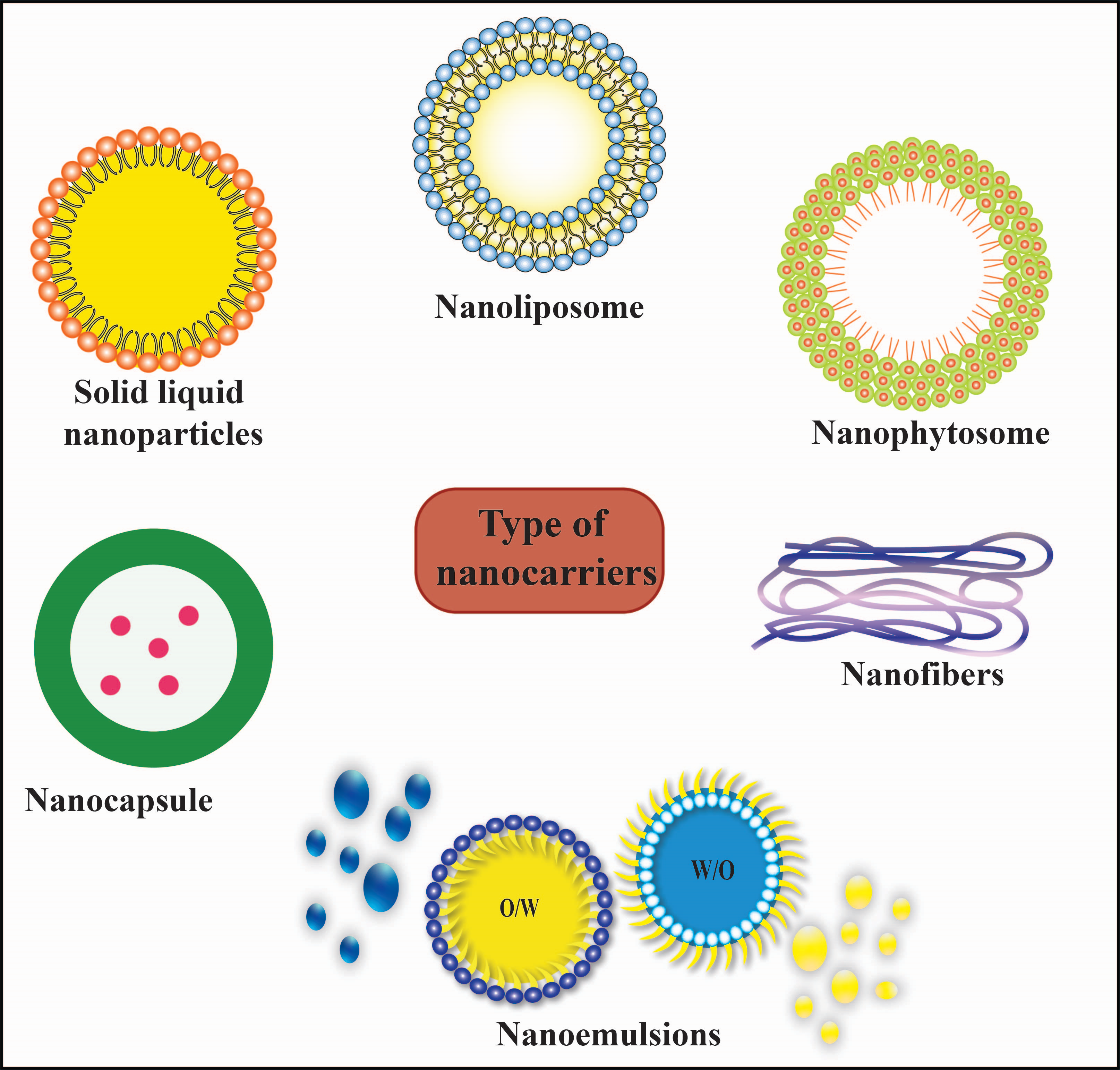


**Figure S1.** Various nanocarriers for the delivery of antioxidant compounds.


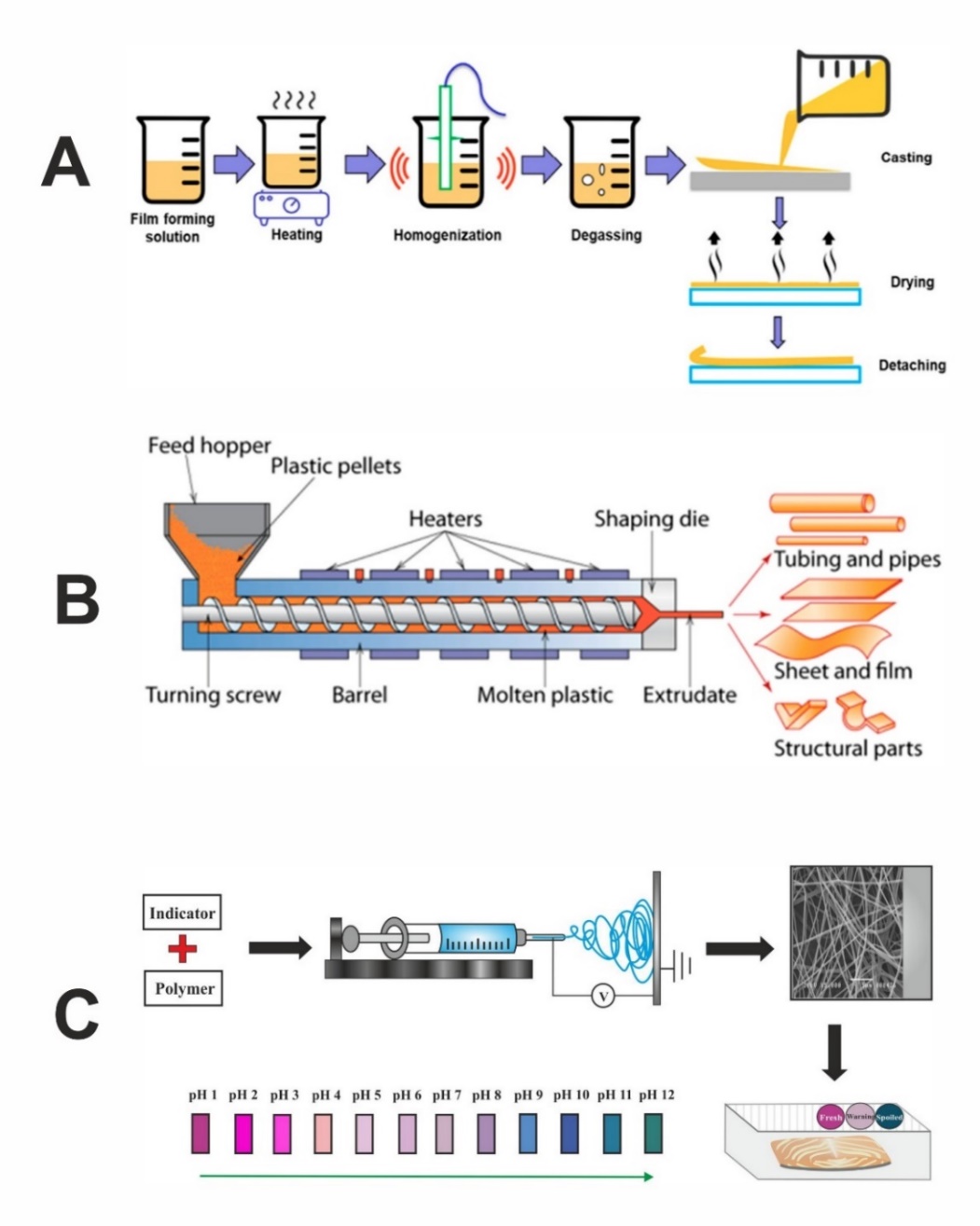


**Figure S2.** The most common methods of food packaging film synthesis: (A): Casting, (B): Extrusion, (C): Electrospinning.
